# Serum osteoprotegerin levels and their association with preeclampsia severity: a case-control study

**DOI:** 10.1186/s12884-026-08638-9

**Published:** 2026-02-02

**Authors:** Ahmed Mohammed Essam El Din Mansour, Ahmed Ramy Mohamed Ramy, Rawan Mahmoud Mohamed Metwaly, Mohamed Ramadan Abd El Latif El Deep, Ahmed Nagy Shaker, Mai Ahmed Ebeid, Mohamed Saeed Khallaf

**Affiliations:** 1 Obstetrics and Gynecology Department, Faculty of Medicine, Ain Shams University, Cairo, Egypt; 2 Clinical Pathology Department, Faculty of Medicine, Ain Shams University, Cairo, Egypt; 3 Obstetrics and Gynecology Department, Faculty of Medicine, 6th of October University, Giza, Egypt; 4 Obstetrics and Gynecology Department, Faculty of Medicine, Kafr El-Sheikh University, Kafr El-Sheikh, Egypt; 5 Clinical Pharmacology Department, Faculty of Medicine, Ain Shams University, Cairo, Egypt

**Keywords:** Preeclampsia, Osteoprotegerin, Biomarker, Pregnancy, Endothelial dysfunction, Fetal growth restriction, ROC curve analysis

## Abstract

**Background:**

Preeclampsia is a hypertensive disorder of pregnancy associated with significant maternal and neonatal morbidity and mortality. Osteoprotegerin (OPG), a glycoprotein involved in vascular homeostasis, has emerged as a potential biomarker for disease severity. This study aimed to evaluate serum OPG levels in pregnant women with and without late-onset preeclampsia and to assess its correlation with disease severity and clinical outcomes.

**Methods:**

This case–control study was conducted at Ain Shams University Maternity Hospital between August 2022 and January 2024. Ninety pregnant women with gestational age ≥ 34 weeks were enrolled and equally divided into three groups: normotensive controls (*n* = 30), mild late-onset preeclampsia (*n* = 30), and severe late-onset preeclampsia (*n* = 30). Clinical assessments, laboratory investigations, and serum OPG levels (measured by ELISA) were compared among groups. Correlation, multivariate regression, and receiver operating characteristic (ROC) curve analyses were performed.

**Results:**

Serum OPG levels were significantly higher in women with late-onset preeclampsia, particularly in the severe group (*p* < 0.0001). Elevated OPG levels were strongly correlated with adverse perinatal outcomes, including fetal growth restriction, preterm delivery, NICU admission, and cesarean delivery. Multivariate regression analysis demonstrated that OPG levels were independently associated with preeclampsia severity and adverse perinatal outcomes. ROC analysis showed excellent diagnostic performance of OPG in identifying severe late-onset preeclampsia (AUC = 0.976), with sensitivity and specificity of 93.3%.

**Conclusion:**

Serum OPG levels are significantly elevated in late-onset preeclampsia and correlate closely with disease severity and adverse maternal and fetal outcomes. OPG shows promise as a clinical biomarker for identifying severe late-onset preeclampsia, although its utility in mild disease appears limited.

**Supplementary Information:**

The online version contains supplementary material available at 10.1186/s12884-026-08638-9.

## Introduction

Preeclampsia is a pregnancy-specific hypertensive disorder that significantly contributes to maternal and perinatal morbidity and mortality worldwide. It affects approximately 2–8% of pregnancies and remains a leading cause of adverse outcomes for both mother and fetus [1]. The incidence of severe forms of preeclampsia has increased markedly in recent decades, underscoring the need for improved diagnostic and prognostic tools. According to the American College of Obstetricians and Gynecologists (ACOG), preeclampsia is characterized by new-onset hypertension (≥ 140/90 mm Hg) after 20 weeks of gestation in previously normotensive women, often accompanied by proteinuria or signs of end-organ dysfunction. Severe preeclampsia involves higher blood pressure thresholds (≥ 160/110 mm Hg), thrombocytopenia, hepatic or renal impairment, pulmonary edema, or neurological symptoms [2, 3]. Despite decades of research, the precise pathophysiological mechanisms underlying preeclampsia remain incompletely understood. The most accepted theory is a two-stage model involving impaired placentation due to insufficient trophoblast invasion and inadequate remodeling of spiral arteries, leading to placental hypoperfusion. This results in systemic endothelial dysfunction, which contributes to the maternal clinical manifestations of the disease. As such, preeclampsia is increasingly viewed as a vascular disorder of pregnancy [4]. In recent years, there has been growing interest in identifying biomarkers that could aid in the early prediction or stratification of preeclampsia severity [5]. One such candidate is osteoprotegerin (OPG), a glycoprotein belonging to the tumor necrosis factor (TNF) receptor superfamily. Originally identified as a regulator of bone turnover, OPG is also involved in vascular homeostasis and endothelial protection. It is expressed in various tissues, including vascular smooth muscle cells, endothelial cells, and the placenta. During pregnancy, serum OPG levels naturally rise, partly due to placental production [5, 6]. Emerging evidence suggests that OPG may play a role in the pathogenesis of preeclampsia. Elevated maternal serum OPG concentrations have been observed in women with preeclampsia, particularly in those with severe disease [5]. Furthermore, genetic polymorphisms in the OPG gene have been associated with vascular diseases such as coronary artery disease, atherosclerosis, and hypertension [7]. These findings support the hypothesis that OPG could serve as a potential biomarker for vascular dysfunction in preeclampsia [8]. Given the potential link between OPG and vascular abnormalities in preeclampsia, further investigation into its clinical utility is warranted. This study aimed to evaluate serum OPG levels in pregnant women with and without preeclampsia and to explore its association with disease severity.

## Patients and methods

**Fig. 1:**
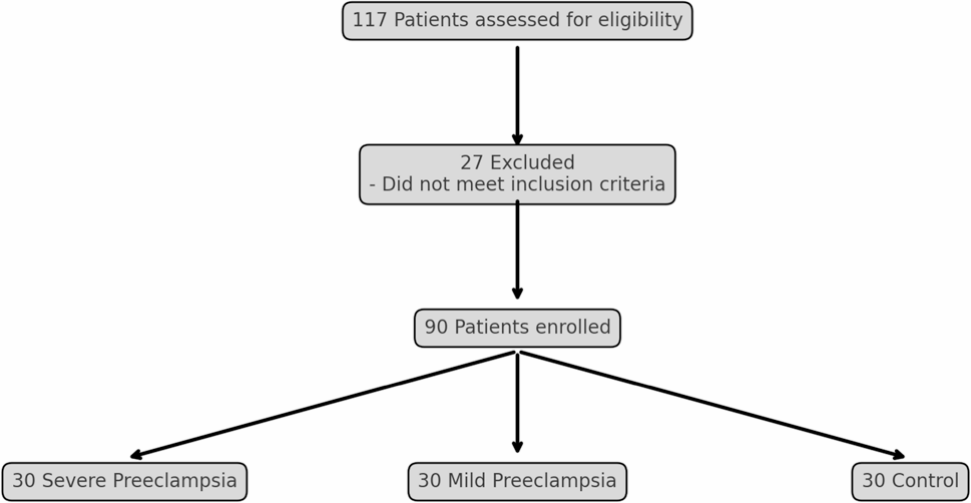
Flow chart of the participants

This case-control study was carried out at Ain Shams University Maternity Hospital over a six-month period from August 2022 to January 2024. A total of 117 pregnant women were initially assessed for eligibility. Twenty-seven women were excluded during the screening process as they did not meet one or more of the predefined inclusion criteria. The reasons for exclusion included: gestational age < 34 weeks at the time of presentation (*n* = 11), history of chronic medical illness such as pre-pregnancy hypertension or renal disease (*n* = 7), current smoking (*n* = 4), use of medications other than routine prenatal supplements (*n* = 4), and detection of fetal structural anomalies on ultrasound evaluation (*n* = 1). After screening, 90 eligible pregnant women with gestational age ≥ 34 weeks were enrolled and equally allocated into three groups (30 per group). No participants were excluded after enrollment. The first group (Group A) comprised 30 normotensive women with uncomplicated pregnancies and served as the control. Group B included 30 participants diagnosed with mild late-onset preeclampsia, while Group C consisted of 30 women with severe late-onset preeclampsia (Fig. 1). The diagnosis of preeclampsia and its classification were based on the most recent guidelines issued by the American College of Obstetricians and Gynecologists [2, 3]. Severe cases were identified by the presence of systolic blood pressure ≥ 160 mmHg or diastolic pressure ≥ 110 mmHg on two separate readings at least four hours apart while at rest, along with at least one of the following: platelet count < 100,000/µL, liver enzyme levels at least twice the upper normal limit, persistent right upper quadrant pain, renal impairment (serum creatinine > 1.1 mg/dL or a doubling of baseline values), pulmonary edema, or new-onset cerebral or visual disturbances. A stratified purposive nonprobability sampling method was employed to recruit participants equally into control, mild preeclampsia, and severe preeclampsia groups based on ACOG diagnostic criteria [2, 3]. Because the inclusion criteria required a gestational age ≥ 34 weeks, all preeclampsia cases in this study represent late-onset preeclampsia according to ACOG definition [3]. The sample size was determined based on prior findings by Oikonomou et al. [5], who reported significantly lower serum osteoprotegerin (OPG) levels in healthy term pregnancies compared to preeclamptic cases (3.81 ± 0.71versus 5.28 ± 1.64 respectively). Using PASS software version 15.0.10, a total of 90 participants (30 per group) was deemed sufficient to achieve 100% power at a significance level of 0.05. Ethical approval was obtained from the Institutional Review Board of the Faculty of Medicine number (MS/396/2022), Ain Shams University, and all participants provided written informed consent before enrollment. Eligible women were between 20 and 35 years of age, had viable singleton pregnancies beyond 34 weeks of gestation, and no previous history of chronic illness. Women were excluded if they had a history of pre-pregnancy hypertension, were smokers, were taking medications other than standard prenatal supplements (iron and folic acid), or if the fetus showed any malformations on ultrasound.

All participants underwent a thorough clinical evaluation including detailed medical and obstetric history, physical examination with assessment of vital signs, and measurement of body mass index (BMI). Routine laboratory investigations were conducted, including complete blood count, renal function tests (serum urea and creatinine), and liver enzyme levels (ALT and AST). Ultrasound imaging was performed to confirm gestational age, evaluate fetal well-being, and rule out anomalies or complications such as retroplacental hematoma or placental abruption.

Peripheral venous blood samples were collected immediately prior to placental delivery. Serum was separated by centrifugation, aliquoted into sterile polypropylene tubes, and stored at − 80 °C until analysis. Blood samples intended for OPG measurement were collected immediately postpartum, ensuring consistency in sampling conditions. Serum osteoprotegerin (OPG) concentrations were determined using a commercially available sandwich enzyme-linked immunosorbent assay (ELISA) kit (Elabscience^®^, Wuhan, China; Catalog No. E-EL-H1341). The assay has a sensitivity of 0.1 ng/mL and a standard curve range of 0.16–10 ng/mL. All reagents and samples were equilibrated to room temperature prior to use, and serial dilutions of recombinant OPG standards were prepared according to the manufacturer’s instructions. Serum samples were analyzed without dilution (neat). In brief, 100 µL of undiluted serum samples and standards were added in duplicate to 96-well microplates pre-coated with anti-human OPG capture antibodies. After incubation and washing, biotin-conjugated anti-OPG detection antibodies were added, followed by HRP-streptavidin. Color development was achieved using TMB substrate, producing a colorimetric change proportional to OPG concentration. The reaction was stopped with an acidic stop solution, and optical density was measured at 450 nm using a microplate reader. A standard curve was generated for each assay run, and sample OPG concentrations were calculated from the corresponding curve. All steps were performed in accordance with the manufacturer’s protocol to ensure accuracy and reproducibility.

Participants were followed for 48 h after delivery to monitor maternal and neonatal outcomes, including blood pressure changes, liver and renal function, fetal birth weight, and any immediate postpartum complications. The primary outcome of the study was the serum OPG level and its diagnostic performance in identifying preeclampsia. Secondary outcomes included the relationship between serum OPG and preeclampsia severity, as well as any associated maternal complications (e.g., placental abruption, HELLP syndrome, preterm labor, organ failure) and neonatal complications (e.g., low birth weight, NICU admission, or neurological impairments). All data were processed using Microsoft Excel 2019 and analyzed with IBM SPSS software version 25.0. Descriptive statistics were reported as means and standard deviations for continuous variables and as frequencies and percentages for categorical data. Intergroup comparisons were performed using the Chi-square test for categorical variables and the Kruskal-Wallis H test for non-normally distributed continuous variables. Receiver operating characteristic (ROC) curves were constructed to evaluate the diagnostic accuracy of serum OPG, including determination of optimal cutoff values, sensitivity, specificity, and area under the curve (AUC). A p-value of < 0.05 was considered statistically significant, with values < 0.01 and < 0.001 indicating moderate and strong significance, respectively.

## Results

**Fig. 2:**
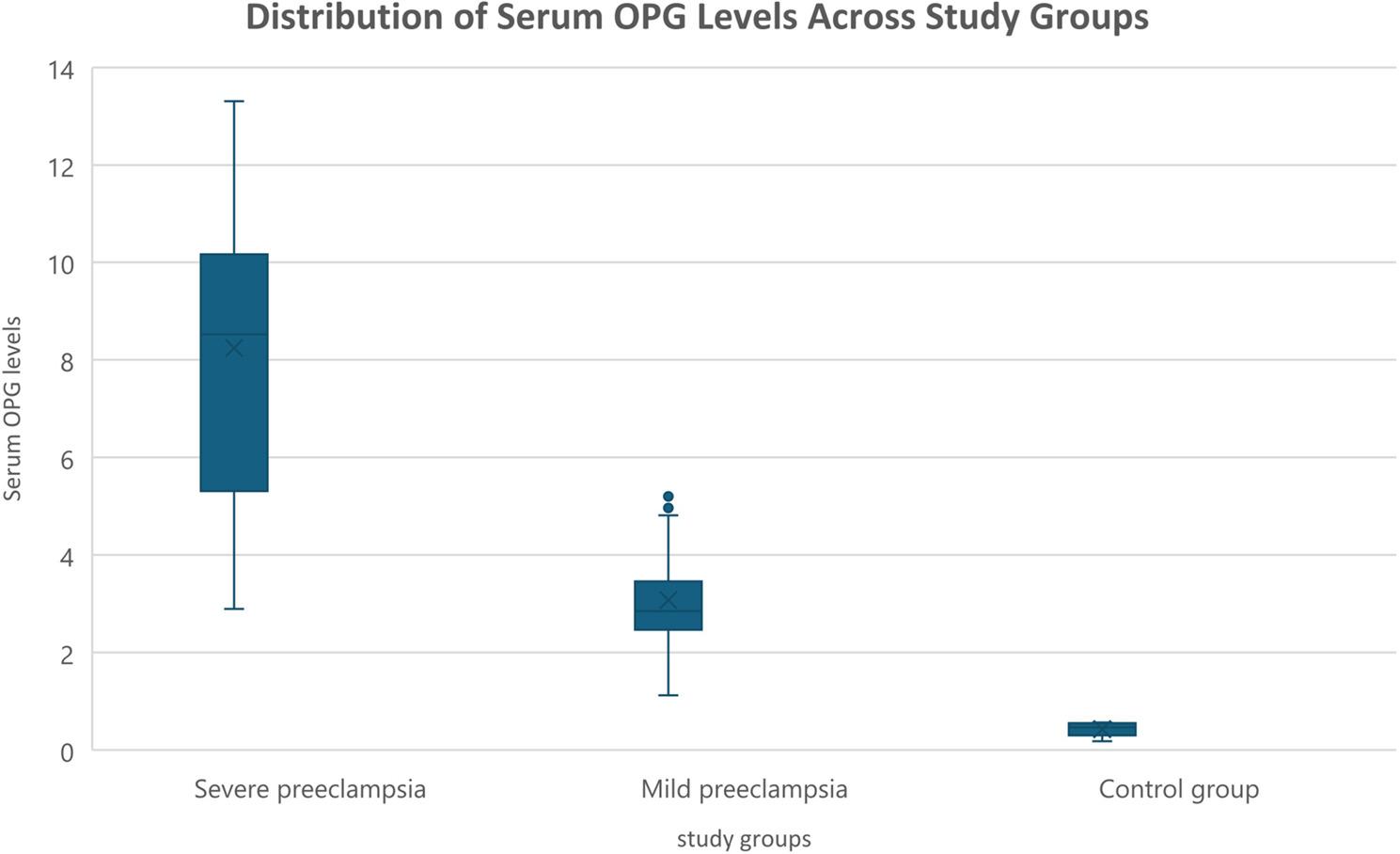
Distribution of serum OPG levels across study groups

**Fig. 3:**
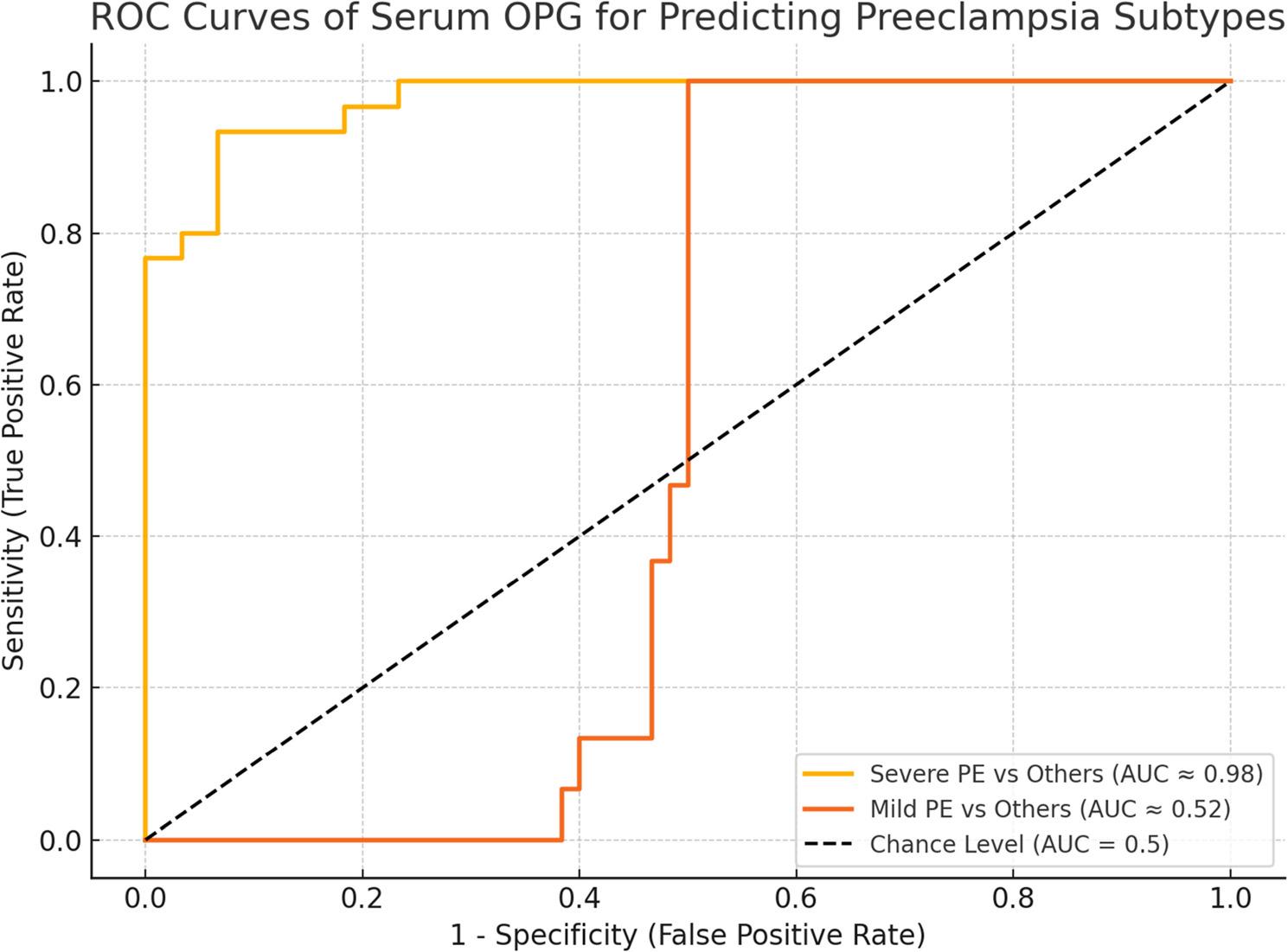
ROC curve of serum OPG for predicting severity of preeclampsia

Table 1 shows that baseline maternal characteristics (age, BMI, gravidity, parity, previous miscarriage, and gestational age at delivery) did not differ significantly among severe preeclampsia, mild preeclampsia, and control groups (all *p* > 0.05). In contrast, systolic and diastolic blood pressures were significantly higher in preeclamptic women, particularly in severe cases (*p* < 0.001). Albuminuria was more frequent in preeclampsia (76.7% in severe vs. 16.7% in controls, *p* < 0.0001). Women with severe preeclampsia delivered earlier, had neonates with lower birth weights, and exhibited markedly higher serum OPG levels (all *p* < 0.0001) (Fig. 2). Cesarean section was most frequent in severe cases (90%, *p* < 0.0005). Adverse neonatal outcomes, including FGR, oligohydramnios, and NICU admissions, were significantly higher in the severe group (*p* < 0.01), while RDS showed a borderline association (*p* = 0.071). Table 2 demonstrates that across all participants, OPG levels were inversely correlated with birth weight and gestational age at delivery, and positively correlated with FGR, NICU admission, cesarean delivery, and albuminuria. No significant correlations were observed with maternal age, BMI, blood pressure, or obstetric history. Focusing on preeclampsia (Table 3), OPG correlated strongly with systolic (*r* = 0.645, *p* < 0.0001) and diastolic blood pressures (*r* = 0.719, *p* < 0.0001) and negatively with birth weight (*r* = − 0.545, *p* < 0.0001). Moderate associations were found with cesarean delivery (*r* = 0.395, *p* = 0.002) and FGR (*r* = 0.305, *p* = 0.018). Other maternal and perinatal variables were not significant. In the control group, no variable correlated significantly with OPG levels. Multivariable regression (Table 4) confirmed that OPG levels were independently higher in mild (β = +2.685, *p* = 0.023) and severe preeclampsia (β = +7.211, *p* < 0.001) compared to controls. Higher MAP was positively associated with OPG (β = +0.033, *p* = 0.031), while oligohydramnios (β = −1.527, *p* = 0.001) and neonatal RDS (β = −1.157, *p* = 0.019) were linked to lower OPG, and NICU admission was associated with higher levels (β = +1.347, *p* = 0.007). The final model explained nearly 80% of the variance in OPG (adjusted R² = 0.794, *p* < 0.001). Table 5 presents the diagnostic performance of OPG. For severe preeclampsia, OPG showed excellent discrimination (AUC = 0.976, 95% CI: 0.937–1.015) with 93.3% sensitivity and specificity at a cutoff of 4.266 ng/mL, yielding a high positive likelihood ratio (14.0) and low negative likelihood ratio (0.071). Conversely, OPG was not useful in distinguishing mild preeclampsia (AUC = 0.524) (Fig. 3).

**Table 1:** Maternal, perinatal, and clinical outcomes among study groups (*N* = 30 per group)

Variable	Severe Preeclampsia (*N* = 30)	Mild Preeclampsia (*N* = 30)	Control Group (*N* = 30)	*P* value
Age				0.123
• Median (Q3–Q1)	30.50 (36.25–26.75)	35.00 (37.00–26.00)	29.00 (33–25.75)	
• Mean ± SD	31.10 ± 5.40	32.50 ± 5.98	29.53 ± 4.35	
BMI (kg/m²)				0.289
• Median (Q3–Q1)	29.45 (33.24–26.65)	27.86 (30.33–24.83)	27.62 (31.38–25.24)	
• Mean ± SD	29.46 ± 3.94	27.94 ± 3.63	28.32 ± 4.01	
Gestational Age (weeks)				0.414*
• Median (Q3–Q1)	37.5 (38.21–36.14)	37.00 (37.90–35.22)	37.07 (37.86–35.11)	
• Mean ± SD	37.18 ± 1.63	36.74 ± 1.66	36.67 ± 1.57	
Gravidity				0.603
• Median (Q3–Q1)	3.50 (4.00–2.25)	3.00 (4.00–2.00)	3.50 (4.00–2.00)	
• Mean ± SD	3.37 ± 1.30	3.03 ± 1.35	3.03 ± 1.56	
Parity				0.491
• Median (Q3–Q1)	1.00 (2.00–0.00)	1.00 (1.75–0.00)	0.00 (2.00–0.00)	
• Mean ± SD	1.23 ± 1.14	0.93 ± 1.08	0.97 ± 1.22	
Previous Miscarriages				0.997
• Median (Q3–Q1)	1.00 (2.00–0.00)	0.50 (2.00–0.00)	1.00 (2.00–0.00)	
• Mean ± SD	1.13 ± 1.25	1.10 ± 1.32	1.07 ± 1.31	
SBP (mmHg)				< 0.001
• Median (Q3–Q1)	183.00 (191.25–172.25)	149.50 (155.00–144.75)	123.50 (129.75–116.50)	
• Mean ± SD	182.40 ± 11.49	150.00 ± 6.10	123.40 ± 7.99	
DBP (mmHg)				< 0.001
• Median (Q3–Q1)	116.00 (118.75–113.00)	100.00 (105.00–94.00)	75.00 (80.00–71.25)	
• Mean ± SD	115.57 ± 3.30	99.57 ± 6.46	76.03 ± 5.03	
Albumin				< 0.0001
• Positive	23 (76.7%)	19 (63.3%)	5 (16.7%)	
• Negative	7 (23.3%)	11 (36.7%)	25 (83.3%)	
Gestational Age at Delivery (weeks)				< 0.0001
• Mean ± SD	37.27 ± 1.62	38.19 ± 0.77	39.23 ± 0.55	
• Median (Q3–Q1)	37.57 (38.33–36.25)	38.07 (38.47–37.57)	39.29 (39.71–38.71)	
Birth Weight (g)				< 0.0001
• Mean ± SD	2050.5 ± 321.55	2671 ± 349.01	3087.33 ± 416.71	
• Median (Q3–Q1)	2015 (2332.25–1763.5)	2743.5 (3023–2399)	3135.5 (3394.5–2752.5)	
OPG ELISA Level (ng/mL)				< 0.0001
• Mean ± SD	8.25 ± 3.10	3.07 ± 0.94	0.33 ± 0.18	
• Median (Q3–Q1)	8.52 (10.16–5.31)	2.84 (3.45–2.46)	0.32 (0.41–0.19)	
Mode of Delivery				< 0.0005
• Cesarean	27 (90%)	14 (46.7%)	11 (36.7%)	
• Vaginal (NVD)	3 (10%)	16 (53.3%)	19 (63.3%)	
Fetal Growth Restriction (FGR)				< 0.0001
• Yes	22 (73.3%)	11 (36.7%)	4 (13.3%)	
• No	8 (26.7%)	19 (63.3%)	26 (86.7%)	
Oligohydramnios				0.003
• Yes	16 (53.3%)	5 (16.7%)	6 (20%)	
• No	14 (46.7%)	25 (83.3%)	24 (80%)	
Respiratory Distress Syndrome (RDS)				0.071
• Yes	13 (43.3%)	11 (36.7%)	5 (16.7%)	
• No	17 (56.7%)	19 (63.3%)	25 (83.3%)	
NICU Admission				< 0.0001
• Yes	22 (73.3%)	15 (50%)	5 (16.7%)	
• No	8 (26.7%)	15 (50%)	25 (83.3%)	

Continuous variables were tested for normality using the Shapiro–Wilk test and are presented as mean ± SD, and median (Q3–Q1). Normally distributed data were compared using one-way ANOVA with Tukey’s HSD post-hoc test, and non-normally distributed data using the Kruskal–Wallis test. Categorical variables are expressed as number (percentage) and compared using the Chi-square test. A p-value < 0.05 was considered statistically significant

**Table 2:** Spearman correlation between OPG levels and clinical variables

Variable	Spearman’s rho	95% CI for rho	*R*²	95% CI for *R*²	*P*-value	Test Used
Age (years)	0.111	(–0.098, 0.311)	0.012	(0.010, 0.097)	0.298	Spearman
BMI (kg/m²)	0.105	(–0.104, 0.306)	0.011	(0.011, 0.094)	0.323	Spearman
Gestational age at enrollment	0.139	(–0.068, 0.335)	0.019	(0.004, 0.112)	0.190	Spearman
Gravidity	0.081	(–0.128, 0.283)	0.007	(0.017, 0.080)	0.449	Spearman
Parity	0.128	(–0.081, 0.326)	0.016	(0.007, 0.107)	0.230	Spearman
Previous Miscarriage	0.035	(–0.173, 0.241)	0.001	(0.030, 0.058)	0.740	Spearman
SBP (mmHg)	–0.058	(–0.263, 0.150)	0.003	(0.022, 0.068)	0.589	Spearman
DBP (mmHg)	–0.063	(–0.268, 0.145)	0.004	(0.021, 0.071)	0.558	Spearman
Gestational Age (weeks) at delivery	–0.542	(–0.674, − 0.378)	0.294	(0.143, 0.454)	< 0.001	Spearman
Birth Weight (grams)	–0.706	(–0.797, − 0.585)	0.499	(0.342, 0.635)	< 0.001	Spearman
Albumin (Positive = 1, Negative = 0)	0.384	(0.192, 0.547)	0.147	(0.037, 0.300)	0.0002	Point-Biserial
Mode of Delivery (CS = 1, NVD = 0)	0.431	(0.246, 0.586)	0.186	(0.061, 0.344)	< 0.0001	Point-Biserial
FGR (Yes = 1, No = 0)	0.452	(0.270, 0.603)	0.204	(0.073, 0.363)	< 0.0001	Point-Biserial
RDS (Yes = 1, No = 0)	0.079	(–0.130, 0.282)	0.006	(0.017, 0.079)	0.4578	Point-Biserial
Oligohydramnios (Yes = 1, No = 0)	0.134	(–0.075, 0.332)	0.018	(0.006, 0.110)	0.2090	Point-Biserial
NICU Admission (Yes = 1, No = 0)	0.422	(0.236, 0.579	0.178	(0.056, 0.335)	< 0.0001	Point-Biserial

Spearman’s rank correlation coefficient (ρ) was used to assess monotonic relationships between serum osteoprotegerin (OPG) levels and clinical variables. This non-parametric test was chosen due to the non-normal distribution of several variables and the ordinal nature of parameters such as gravidity and parity. Point-biserial correlation was used for dichotomous binary variables (e.g., albumin, mode of delivery). A p-value < 0.05 was considered statistically significant. Coefficient of determination (R²) was calculated by squaring ρ to estimate the proportion of variance explained

**Table 3:** Correlation between OPG levels and clinical variables (preeclampsia vs. control)

Variable	Preeclampsia (*r*, *p*)	Control (*r*, *p*)
Age (years)	–0.103, *p* = 0.436	0.014, *p* = 0.942
BMI (kg/m²)	0.091, *p* = 0.491	0.224, *p* = 0.233
Gestational age at enrollment	0.172, *p* = 0.188	–0.094, *p* = 0.620
Gravidity	0.093, *p* = 0.481	0.023, *p* = 0.903
Parity	0.099, *p* = 0.452	0.251, *p* = 0.181
Previous Miscarriage	0.055, *p* = 0.676	–0.057, *p* = 0.764
SBP (mmHg)	0.645, *p* < 0.0001	0.172, *p* = 0.363
DBP (mmHg)	0.719, *p* < 0.0001	–0.001, *p* = 0.995
Gestational Age at Delivery (weeks)	–0.178, *p* = 0.174	0.011, *p* = 0.955
Birth Weight (grams)	–0.545, *p* < 0.0001	0.010, *p* = 0.958
Albumin (Positive = 1)	0.080, *p* = 0.545	0.294, *p* = 0.115
Mode of Delivery (CS = 1)	0.395, *p* = 0.002	0.243, *p* = 0.195
FGR (Yes = 1)	0.305, *p* = 0.018	–0.135, *p* = 0.477
RDS (Yes = 1)	–0.120, *p* = 0.361	–0.108, *p* = 0.572
Oligohydramnios (Yes = 1)	0.055, *p* = 0.676	–0.294, *p* = 0.115
NICU Admission (Yes = 1)	0.232, *p* = 0.074	0.083, *p* = 0.663

Spearman’s rank correlation coefficient (r) was applied for continuous or ordinal variables due to non-parametric distributions. Point-biserial correlation was used for dichotomous binary variables (e.g., albumin, mode of delivery). A p-value < 0.05 was considered statistically significant

**Table 4:** Multiple linear regression analysis for predicting serum OPG levels *(reduced model after multicollinearity adjustment)*

Predictor	Coefficient (β)	Standard Error	t-value	*p*-value	Significance
Constant	−2.914	8.727	−0.334	0.739	
Age (years)	−0.037	0.036	−1.018	0.312	
Gestational Age at Enrollment	−0.076	0.171	−0.444	0.658	
BMI (kg/m²)	−0.059	0.049	−1.194	0.236	
MAP (mmHg)	+ 0.033	0.015	+ 2.199	0.031	*
Group: Mild PE vs. Control	+ 2.685	1.158	+ 2.318	0.023	*
Group: Severe PE vs. Control	+ 7.211	1.553	+ 4.642	< 0.001	**
Albumin (Positive)	−0.305	0.448	−0.681	0.498	
Mode of Delivery (NVD)	+ 0.781	0.420	+ 1.858	0.067	
Gestational Age at Delivery	+ 0.445	0.284	+ 1.569	0.121	
Birth Weight (grams)	−0.001	0.001	−1.401	0.165	
Fetal Growth Restriction (Yes)	+ 0.742	0.457	+ 1.624	0.108	
Oligohydramnios (Yes)	−1.527	0.456	−3.349	0.001	**
Respiratory Distress Syndrome	−1.157	0.484	−2.392	0.019	*
NICU Admission (Yes)	+ 1.347	0.486	+ 2.774	0.007	**

Model Summary

• R² = 0.822

• Adjusted R² = 0.794

• F-statistic (overall model) = 29.07

• Model p-value = < 0.001

• Significance thresholds:

o *p* < 0.05 = *

o *p* < 0.01 = **

A multiple linear regression model was developed using serum OPG level as the dependent variable. To avoid multicollinearity, variables with high Variance Inflation Factor (VIF), including parity, gravidity, and diastolic BP, were excluded. Mean Arterial Pressure (MAP) was used instead of SBP/DBP to reflect overall hemodynamic burden. Categorical variables were converted into binary dummy variables. The model was assessed for fit using R², adjusted R², and the F-statistic. A p-value < 0.05 was considered statistically significant

**Table 5:** ROC curve analysis of serum OPG levels for preeclampsia subtypes

Metric	Severe PE vs. Others	Mild PE vs. Others
AUC	0.976	0.524
95% CI (AUC)	[0.937, 1.015]	[0.396, 0.652]
Optimal Cutoff (OPG)	4.266 ng/mL	1.123 ng/mL
Sensitivity	93.3%	100%
Specificity	93.3%	50%
Positive Predictive Value	87.5%	40.0%
Negative Predictive Value	96.2%	100%
Positive Likelihood Ratio	14.00	2.00
Negative Likelihood Ratio	0.071	0.00
Accuracy	93.3%	60.5%

Receiver Operating Characteristic (ROC) curve analysis was performed using serum OPG levels to evaluate the test’s ability to discriminate between preeclampsia subtypes and other groups. The area under the curve (AUC) with 95% confidence intervals was used to assess model discrimination. The Youden index determined the optimal cutoff point. Diagnostic accuracy was further evaluated using sensitivity, specificity, positive predictive value (PPV), negative predictive value (NPV), positive likelihood ratio (LR+), negative likelihood ratio (LR−), and overall classification accuracy

## Discussion

Preeclampsia (PE) is a hypertensive disorder of pregnancy known for its complex etiology and significant contribution to maternal and perinatal morbidity and mortality worldwide [9]. Although its precise pathophysiology remains incompletely understood, accumulating evidence implicates endothelial dysfunction, inflammation, and abnormal placentation as central mechanisms [10]. In recent years, osteoprotegerin (OPG), a member of the tumor necrosis factor receptor superfamily, has emerged as a potential biomarker due to its roles in vascular biology, inflammation modulation, and endothelial protection [6]. This study examined serum osteoprotegerin (OPG) levels in pregnant women with varying severities of late-onset preeclampsia compared to normotensive controls, revealing a significant increase in OPG levels among those with preeclampsia, particularly in severe cases. The observed dose–response relationship suggests a positive correlation between OPG expression and disease severity, likely reflecting the extent of vascular dysfunction. These findings are consistent with prior evidence indicating OPG’s potential protective role in vascular homeostasis by inhibiting endothelial apoptosis and arterial calcification [11]. Notably, the association remained robust after adjusting for clinical and obstetric confounders, with the regression model accounting for approximately 80% of the variability in OPG levels. Collectively, these results highlight the potential utility of OPG as a biomarker for assessing disease severity and guiding risk stratification in preeclampsia.

Although previous study [6], including work in non-pregnant cardiovascular and hypertensive populations, have linked OPG to vascular dysfunction, these findings cannot be directly extrapolated to the pregnancy-specific and placental-driven pathophysiology of preeclampsia. The present study adds several novel contributions to the existing literature. First, we focused exclusively on late-onset preeclampsia (≥ 34 weeks), a clinically and biologically distinct phenotype, and demonstrated a clear and graded increase in OPG levels across normotensive, mild, and severe disease categories using strict ACOG criteria. Second, we comprehensively evaluated associations between serum OPG and a broad spectrum of maternal and perinatal outcomes, including fetal growth restriction, gestational age at delivery, NICU admission, and mode of delivery—outcomes that have not been systematically explored in prior studies. Third, and importantly, we assessed the diagnostic performance of OPG and demonstrated excellent discriminatory power for identifying severe late-onset preeclampsia through ROC curve analysis. Together, these findings extend current knowledge by establishing OPG not only as a marker of vascular dysfunction but also as a clinically relevant indicator of disease severity and adverse pregnancy outcomes in late-onset preeclampsia.

Although the three study groups were comparable with respect to important maternal and obstetric baseline characteristics—including age, BMI, gravidity, parity, and miscarriage history—differences in perinatal outcomes were expected and reflect the underlying disease severity rather than pre-existing group differences. Consistent with the diagnostic criteria for preeclampsia, both systolic and diastolic blood pressures and the frequency of albuminuria were significantly higher in the preeclamptic groups, particularly among women with severe disease. These clinical differences were accompanied by the anticipated gradient in perinatal outcomes, with the severe preeclampsia group demonstrating earlier gestational age at delivery and markedly lower birth weight [12, 13]. Importantly, the absence of significant differences in demographic or obstetric characteristics supports that the observed elevations in serum OPG levels were unlikely to be driven by baseline confounders. Instead, the strong correlations between higher OPG concentrations and adverse perinatal outcomes—including lower gestational age at delivery, reduced birth weight, increased fetal growth restriction, higher cesarean delivery rates, and greater NICU admission—reinforce the potential role of OPG as a biomarker reflecting the degree of placental dysfunction and fetal compromise [8, 14]. Given the placental origin of OPG and its expression in cytotrophoblasts and syncytiotrophoblasts [8], this finding is biologically plausible and clinically meaningful. Interestingly, OPG demonstrated strong positive correlations with both systolic and diastolic blood pressures specifically in preeclampsia cases (ρ = 0.645 and 0.719, respectively), but not in the control group. This suggests that OPG may function as a disease-specific vascular biomarker, rather than reflecting general hemodynamic changes in normal pregnancies. Previous studies have similarly found OPG to be associated with vascular endothelial dysfunction and inflammatory states in conditions such as hypertension and coronary artery disease [6, 15]. Shen et al. similarly demonstrated upregulated OPG expression—both protein and mRNA—in placental tissues from preeclamptic women, with the highest levels observed in severe cases. This is consistent with our current findings where serum OPG levels positively correlated with systolic and diastolic blood pressure, and inversely with neonatal birth weight, suggesting a role in disease progression and adverse outcomes [8]. Moreover, albuminuria was significantly more common among preeclamptic groups in our cohort, reflecting endothelial dysfunction and glomerular damage. Although a subset of severe cases presented without overt albuminuria, hypoalbuminemia may still reflect systemic protein loss or impaired hepatic function, which has been linked to poor maternal outcomes in PE [16]. Regarding perinatal outcomes, also there was statistically significant differences were observed across groups in our study. Previous research consistently links preeclampsia to adverse neonatal outcomes, including intrauterine growth restriction (IUGR), preterm birth, and stillbirth [17]. OPG levels were inversely associated with birth weight and gestational age at delivery, suggesting a strong relationship between elevated OPG and poor fetal growth and prematurity. Moderate positive correlations were found with FGR, NICU admission, cesarean delivery, and albuminuria—further supporting the relevance of OPG to disease severity in preeclampsia echoes the findings of Shen et al. [8] and suggests that OPG may be involved in placental insufficiency and fetal growth restriction. Furthermore, ROC curve analysis revealed that serum OPG had excellent diagnostic accuracy for distinguishing severe preeclampsia from mild or normotensive pregnancies, with an AUC of 0.976 and both sensitivity and specificity reaching 93.3%. This finding holds promise for its integration into clinical algorithms for identifying women at highest risk of complications and allocating resources accordingly. However, its utility in detecting mild preeclampsia was limited (AUC = 0.524), indicating that OPG is more effective as a marker of severity rather than for early or low-grade disease detection. The potential mechanisms underlying OPG upregulation in preeclampsia remain to be fully elucidated. OPG is known to increase progressively through pregnancy, peaking in the third trimester [18], and may be upregulated as a compensatory response to endothelial damage. Its expression pattern mirrors that of other placental hormones implicated in PE pathogenesis, such as leptin, IGF-1, and adiponectin [19–21]. It is also possible that OPG plays an active role in modulating placental vascular tone, inflammatory signaling, or oxidative stress responses, although this remains speculative. Contrary to findings in the preeclamptic cohort, no statistically significant correlations between OPG and clinical outcomes were observed in the normotensive control group. This lack of association further strengthens the specificity of OPG as a pathophysiological and diagnostic marker for preeclampsia. It also reinforces the hypothesis that elevated OPG reflects disease-specific processes such as endothelial injury and impaired placental perfusion, rather than being a non-specific pregnancy-related change.

It is important to emphasize that the present study exclusively included women with a gestational age of ≥ 34 weeks and therefore specifically reflects the pathophysiology and clinical spectrum of late-onset preeclampsia. Early-onset preeclampsia (< 34 weeks), which is characterized by more profound placental dysfunction and distinct biological mechanisms, was not evaluated in this cohort. Consequently, the observed associations between serum osteoprotegerin levels and disease severity should not be extrapolated to early-onset preeclampsia.

This study demonstrates that serum osteoprotegerin (OPG) levels are significantly higher in women with late-onset preeclampsia compared with normotensive pregnancies and increase in parallel with disease severity. Elevated OPG concentrations were associated with severe clinical phenotypes and adverse maternal and perinatal outcomes, including fetal growth restriction, lower gestational age at delivery, and increased NICU admission. However, because serum OPG was measured at the time of delivery, the present findings do not support its use for prediction, early risk stratification, or real-time clinical decision-making during pregnancy. Rather, the observed associations suggest that OPG reflects established disease severity in late-onset preeclampsia. The relationship between higher OPG levels and adverse outcomes is consistent with known features of advanced disease, such as endothelial dysfunction and placental insufficiency, but causality cannot be inferred from this study design. Taken together, these results indicate that OPG may serve as a biochemical correlate of disease severity in late-onset preeclampsia, rather than a clinical prognostic or management tool. Further longitudinal studies with antenatal sampling are required to determine whether OPG has any role in early disease characterization or prediction of outcomes earlier in pregnancy.

## Conclusion

Our findings show that serum osteoprotegerin (OPG) levels are significantly elevated in late-onset preeclampsia, particularly in severe cases, and are associated with adverse maternal and perinatal outcomes. Because OPG was measured at delivery, these results indicate an association with established disease severity rather than predictive or clinical utility. Further longitudinal studies with antenatal assessment are needed to clarify the potential role of OPG earlier in pregnancy.

### Strengths and limitations

This study has several strengths that enhance its scientific and clinical value. First, the inclusion of well-matched study groups with no significant differences in maternal age, BMI, gravidity, parity, or miscarriage history minimizes potential confounding and supports the validity of the observed associations. Second, stratifying preeclampsia cases into mild and severe categories allowed for clear evaluation of the dose–response relationship between serum osteoprotegerin (OPG) levels and disease severity. Third, the use of comprehensive statistical analyses—including correlation coefficients, multiple linear regression, and ROC curve analysis—provided robust insights into the diagnostic and clinical utility of OPG. The study also evaluated a broad range of maternal and fetal outcomes, enabling a detailed assessment of OPG’s associations with adverse perinatal events such as fetal growth restriction, preterm birth, and NICU admission. Importantly, the study demonstrated excellent diagnostic accuracy for OPG in identifying severe preeclampsia, highlighting its potential as a clinically relevant biomarker.

However, several limitations should be acknowledged. The case–control design precludes assessment of causality or temporal changes in OPG levels across gestation, and the single-center setting with a relatively modest sample size may limit generalizability. Additionally, placental tissue analysis was not performed, preventing evaluation of local OPG expression and related mechanistic pathways. While baseline demographic and obstetric characteristics were matched, other potential confounders—such as vitamin D status, calcium metabolism, and inflammatory markers—were not assessed. OPG measurements were obtained only at delivery, without longitudinal or postpartum follow-up. Importantly, because the inclusion criteria required a gestational age of ≥ 34 weeks, all preeclampsia cases in this cohort represent late-onset disease according to ACOG and ISSHP definitions. Early-onset preeclampsia (< 34 weeks), which is known to have distinct pathophysiology and more severe placental involvement, was not captured; this should be considered when interpreting the applicability of our findings. Finally, although serum OPG demonstrated strong discriminatory ability for severe preeclampsia, it was not effective in identifying mild cases, which limits its utility as a broad screening tool.

## Supplementary Information

Supplementary Material 1.

## Data Availability

The datasets generated and/or analyzed during the current study are included within the published article and its supplementary files. Additional data may be available from the corresponding author on reasonable request.
