# Supplementary material for: Serum osteoprotegerin levels and their association with preeclampsia severity: a case-control study
Source: BMC Pregnancy Childbirth. 2026 Feb 2;26:196. doi: 10.1186/s12884-026-08638-9 (PMC12930896; doi:10.1186/s12884-026-08638-9)
Supplement: Supplementary file 1 — Supplementary Material 1. [file 12884_2026_8638_MOESM1_ESM.docx]

**Supp Table 1. Maternal, Perinatal, and Clinical Outcomes Among Study Groups (N = 30 per group)**

| Variable | Severe Preeclampsia (N=30) | Mild Preeclampsia (N=30) | Control Group (N=30) | P value |
| --- | --- | --- | --- | --- |
| Age |  |  |  | 0.123 |
| • Median (Q3–Q1) | 30.50 (36.25–26.75) | 35.00 (37.00–26.00) | 29.00 (33–25.75) |  |
| • Mean ± SD | 31.10 ± 5.40 | 32.50 ± 5.98 | 29.53 ± 4.35 |  |
| • Range | 23–40 | 22–40 | 22–40 |  |
| BMI (kg/m²) |  |  |  | 0.289 |
| • Median (Q3–Q1) | 29.45 (33.24–26.65) | 27.86 (30.33–24.83) | 27.62 (31.38–25.24) |  |
| • Mean ± SD | 29.46 ± 3.94 | 27.94 ± 3.63 | 28.32 ± 4.01 |  |
| • Range | 23.09–34.94 | 22.30–34.37 | 22.08–34.95 |  |
| Gestational Age (weeks) |  |  |  | 0.414* |
| • Median (Q3–Q1) | 37.5 (38.21–36.14) | 37.00 (37.90–35.22) | 37.07 (37.86–35.11) |  |
| • Mean ± SD | 37.18 ± 1.63 | 36.74 ± 1.66 | 36.67 ± 1.57 |  |
| • Range | 34–39 | 34–39.57 | 34.14–39.29 |  |
| Gravidity |  |  |  | 0.603 |
| • Median (Q3–Q1) | 3.50 (4.00–2.25) | 3.00 (4.00–2.00) | 3.50 (4.00–2.00) |  |
| • Mean ± SD | 3.37 ± 1.30 | 3.03 ± 1.35 | 3.03 ± 1.56 |  |
| • Range | 1–5 | 1–5 | 1–5 |  |
| G1  G2  G3  G4  G5 | 3 (10%)  5 (16.67%)  7 (23.33%)  8 (26.67%)  7 (23.33%) | 5 (16.67%)  7 (23.33%)  4 (13.33%)  10 (33.33%)  4 (13.33%) | 7 (23.33%)  7 (23.33%)  1 (3.33%)  8 (26.67%)  7 (23.33%) | 0.450 |
| Parity |  |  |  | 0.491 |
| • Median (Q3–Q1) | 1.00 (2.00–0.00) | 1.00 (1.75–0.00) | 0.00 (2.00–0.00) |  |
| • Mean ± SD | 1.23 ± 1.14 | 0.93 ± 1.08 | 0.97 ± 1.22 |  |
| • Range | 0–3 | 0–3 | 0–3 |  |
| P0  P1  P2  P3 | 11 (36.67%)  6 (20%)  8 (26.67%)  5 (16.67%) | 14 (46.67%)  8 (26.67%)  4 (13.33%)  4 (13.33%) | 16 (53.33%)  5 (16.67%)  3 (10%)  6 (20%) | 0.561 |
| Previous Miscarriages |  |  |  | 0.997 |
| • Median (Q3–Q1) | 1.00 (2.00–0.00) | 0.50 (2.00–0.00) | 1.00 (2.00–0.00) |  |
| • Mean ± SD | 1.13 ± 1.25 | 1.10 ± 1.32 | 1.07 ± 1.31 |  |
| • Range | 0–4 | 0–4 | 0–4 |  |
| M0  M1  M2  M3  M4 | 13 (43.33%)  6 (20%)  7 (23.33%)  2 (6.67%)  2 (6.67%) | 15 (50%)  5 (16.67%)  3 (10%)  6 (20%)  1 (3.33) | 14 (46.67%)  7 (23.33%)  5 (16.67%)  1 (3.33)  3 (10%) | 0.456 |
| SBP (mmHg) |  |  |  | <0.001 |
| • Median (Q3–Q1) | 183.00 (191.25–172.25) | 149.50 (155.00–144.75) | 123.50 (129.75–116.50) |  |
| • Mean ± SD | 182.40 ± 11.49 | 150.00 ± 6.10 | 123.40 ± 7.99 |  |
| • Range | 165–205 | 140–159 | 111–134 |  |
| DBP (mmHg) |  |  |  | <0.001 |
| • Median (Q3–Q1) | 116.00 (118.75–113.00) | 100.00 (105.00–94.00) | 75.00 (80.00–71.25) |  |
| • Mean ± SD | 115.57 ± 3.30 | 99.57 ± 6.46 | 76.03 ± 5.03 |  |
| • Range | 110–120 | 90–109 | 70–85 |  |
| Albumin |  |  |  | <0.0001 |
| - Positive | 23 (76.7%) | 19 (63.3%) | 5 (16.7%) |  |
| - Negative | 7 (23.3%) | 11 (36.7%) | 25 (83.3%) |  |
| Gestational Age at Delivery (weeks) |  |  |  | <0.0001 |
| • Mean ± SD | 37.27 ± 1.62 | 38.19 ± 0.77 | 39.23 ± 0.55 |  |
| • Median (Q3–Q1) | 37.57 (38.33–36.25) | 38.07 (38.47–37.57) | 39.29 (39.71–38.71) |  |
| • Range | 34.14–39.86 | 37.00–40.00 | 38.29–40.29 |  |
| Birth Weight (g) |  |  |  | <0.0001 |
| • Mean ± SD | 2050.5 ± 321.55 | 2671 ± 349.01 | 3087.33 ± 416.71 |  |
| • Median (Q3–Q1) | 2015 (2332.25–1763.5) | 2743.5 (3023–2399) | 3135.5 (3394.5–2752.5) |  |
| • Range | 1555–2765 | 1999–3196 | 2199–3945 |  |
| OPG ELISA Level (ng/mL) |  |  |  | <0.0001 |
| • Mean ± SD | 8.25 ± 3.10 | 3.07 ± 0.94 | 0.33 ± 0.18 |  |
| • Median (Q3–Q1) | 8.52 (10.16–5.31) | 2.84 (3.45–2.46) | 0.32 (0.41–0.19) |  |
| • Range | 2.89–13.31 | 1.12–5.20 | 0.07–0.87 |  |
| Mode of Delivery |  |  |  | <0.0005 |
| - Cesarean | 27 (90%) | 14 (46.7%) | 11 (36.7%) |  |
| - Vaginal (NVD) | 3 (10%) | 16 (53.3%) | 19 (63.3%) |  |
| Fetal Growth Restriction (FGR) |  |  |  | <0.0001 |
| - Yes | 22 (73.3%) | 11 (36.7%) | 4 (13.3%) |  |
| - No | 8 (26.7%) | 19 (63.3%) | 26 (86.7%) |  |
| Oligohydramnios |  |  |  | 0.003 |
| - Yes | 16 (53.3%) | 5 (16.7%) | 6 (20%) |  |
| - No | 14 (46.7%) | 25 (83.3%) | 24 (80%) |  |
| Respiratory Distress Syndrome (RDS) |  |  |  | 0.071 |
| - Yes | 13 (43.3%) | 11 (36.7%) | 5 (16.7%) |  |
| - No | 17 (56.7%) | 19 (63.3%) | 25 (83.3%) |  |
| NICU Admission |  |  |  | <0.0001 |
| - Yes | 22 (73.3%) | 15 (50%) | 5 (16.7%) |  |
| - No | 8 (26.7%) | 15 (50%) | 25 (83.3%) |  |

**Supp Table 2: Correlation Between OPG Levels and Clinical Variables for preeclampsia groups (mild and severe)**

| Variable | Correlation Coefficient (r) | 95% CI for r | R² | 95% CI for R² | P-value | Test Used |
| --- | --- | --- | --- | --- | --- | --- |
| Age (years) | –0.103 | (–0.347, 0.155) | 0.011 | (0.024, 0.121) | 0.436 | Spearman |
| BMI (kg/m²) | 0.091 | (–0.167, 0.337) | 0.008 | (0.028, 0.113) | 0.491 | Spearman |
| Gestational age at enrollment | 0.172 | (–0.085, 0.408) | 0.029 | (0.0072, 0.166) | 0.188 | Spearman |
| Gravidity | 0.093 | (–0.165, 0.339) | 0.009 | (0.027, 0.115) | 0.481 | Spearman |
| Parity | 0.099 | (–0.159, 0.344) | 0.010 | (0.025, 0.118) | 0.452 | Spearman |
| Previous Miscarriage | 0.055 | (–0.202, 0.305) | 0.003 | (0.041, 0.093) | 0.676 | Spearman |
| SBP (mmHg) | 0.645 | (0.468, 0.773) | 0.416 | (0.219, 0.597) | <0.0001 | Spearman |
| DBP (mmHg) | 0.719 | (0.569, 0.823) | 0.517 | (0.323, 0.677) | <0.0001 | Spearman |
| Gestational Age (weeks) | –0.178 | (–0.413, 0.080) | 0.032 | (0.006, 0.171) | 0.174 | Spearman |
| Birth Weight (grams) | –0.545 | (–0.702, –0.338) | 0.297 | (0.114, 0.493) | <0.0001 | Spearman |
| Albumin (Positive = 1) | 0.080 | (–0.178, 0.327) | 0.006 | (0.032, 0.107) | 0.545 | Point-Biserial |
| Mode of Delivery (CS = 1) | 0.395 | (0.157, 0.590) | 0.156 | (0.025, 0.348) | 0.002 | Point-Biserial |
| FGR (Yes = 1) | 0.305 | (0.055, 0.519) | 0.093 | (0.003, 0.269) | 0.018 | Point-Biserial |
| RDS (Yes = 1) | –0.120 | (–0.363, 0.138) | 0.014 | (0.019, 0.132) | 0.361 | Point-Biserial |
| Oligohydramnios (Yes = 1) | 0.055 | (–0.202, 0.305) | 0.003 | (0.041, 0.093) | 0.676 | Point-Biserial |
| NICU Admission (Yes = 1) | 0.232 | (–0.023, 0.459) | 0.054 | (0.001, 0.211) | 0.074 | Point-Biserial |

***Footnote:*** *Spearman’s rank correlation coefficient (ρ) was applied for continuous or ordinal variables due to non-parametric distributions. Point-biserial correlation was used for dichotomous binary variables (e.g., albumin, mode of delivery). Coefficient of determination (R²) was derived by squaring the correlation coefficient to estimate the explained variance. A p-value < 0.05 was considered statistically significant. Confidence intervals were calculated for both ρ/r and R².*

**Supp Table 3: Correlation Between OPG Levels and Clinical Variables (Control Group)**

| Variable | Correlation Coefficient (r) | 95% CI for r | R² | 95% CI for R² | P-value | Test Used |
| --- | --- | --- | --- | --- | --- | --- |
| Age (years) | 0.014 | (–0.348, 0.372) | 0.000 | (0.121, 0.139) | 0.942 | Spearman |
| BMI (kg/m²) | 0.224 | (–0.148, 0.541) | 0.050 | (0.022, 0.293) | 0.233 | Spearman |
| Gestational age at enrollment | –0.094 | (–0.440, 0.275) | 0.009 | (0.076, 0.193) | 0.620 | Spearman |
| Gravidity | 0.023 | (–0.340, 0.380) | 0.001 | (0.115, 0.145) | 0.903 | Spearman |
| Parity | 0.251 | (–0.120, 0.561) | 0.063 | (0.014, 0.314) | 0.181 | Spearman |
| Previous Miscarriage | –0.057 | (–0.409, 0.310) | 0.003 | (0.096, 0.167) | 0.764 | Spearman |
| SBP (mmHg) | 0.172 | (–0.201, 0.501) | 0.030 | (0.040, 0.251) | 0.363 | Spearman |
| DBP (mmHg) | –0.001 | (–0.361, 0.359) | 0.000 | (0.129, 0.130) | 0.995 | Spearman |
| Gestational Age at Delivery (weeks) | 0.011 | (–0.351, 0.370) | 0.000 | (0.123, 0.137) | 0.955 | Spearman |
| Birth Weight (grams) | 0.010 | (–0.352, 0.369) | 0.000 | (0.124, 0.136) | 0.958 | Spearman |
| Albumin (Positive = 1) | 0.294 | (–0.075, 0.591) | 0.086 | (0.006, 0.350) | 0.115 | Point-Biserial |
| Mode of Delivery (CS = 1) | 0.243 | (–0.128, 0.555) | 0.059 | (0.016, 0.308) | 0.195 | Point-Biserial |
| FGR (Yes = 1) | –0.135 | (–0.472, 0.237) | 0.018 | (0.056, 0.223) | 0.477 | Point-Biserial |
| RDS (Yes = 1) | –0.108 | (–0.450, 0.263) | 0.012 | (0.069, 0.203) | 0.572 | Point-Biserial |
| Oligohydramnios (Yes = 1) | –0.294 | (–0.591, 0.075) | 0.086 | (0.006, 0.350) | 0.115 | Point-Biserial |
| NICU Admission (Yes = 1) | 0.083 | (–0.286, 0.430) | 0.007 | (0.082, 0.185) | 0.663 | Point-Biserial |

***Footnote:*** *Spearman's rank correlation coefficient was used to assess the monotonic association between OPG levels and continuous or ordinal variables due to non-normal data distribution. Point-biserial correlation was applied for dichotomous variables. The coefficient of determination (R²) was calculated to express the proportion of variability in OPG explained by each variable. Confidence intervals (95%) were estimated for both the correlation coefficients and R². A p-value < 0.05 was considered statistically significant.*
